# CRISPRa-mediated *FOXP3* gene upregulation in mammalian cells

**DOI:** 10.1186/s13578-019-0357-0

**Published:** 2019-11-21

**Authors:** Vida Forstnerič, Irena Oven, Jernej Ogorevc, Duško Lainšček, Arne Praznik, Tina Lebar, Roman Jerala, Simon Horvat

**Affiliations:** 1 Department of Synthetic Biology and Immunology, National Institute of Chemistry, Ljubljana, Hajdrihova 19, 1000 Ljubljana, Slovenia; 2 Department of Animal Science, Biotechnical Faculty, University of Ljubljana, Groblje 3, 1230 Domžale, Slovenia; 3 EN-FIST Centre of Excellence, Trg Osvobodilne fronte 13, 1000 Ljubljana, Slovenia

**Keywords:** CRISPR, *FOXP3*, Transcription regulation

## Abstract

**Background:**

Forkhead box P3^+^ (*FOXP3*^+^) regulatory T cells (Tregs) are a subset of lymphocytes, critical for the maintenance of immune homeostasis. Loss-of-function mutations of the *FOXP3* gene in animal models and humans results in loss of differentiation potential into Treg cells and are responsible for several immune-mediated inflammatory diseases. Strategies of increasing *FOXP3* expression represent a potential approach to increase the pool of Tregs within the lymphocyte population and may be employed in therapies of diverse autoimmune conditions. In the present study, a dCas9 CRISPR-based method was systematically employed to achieve upregulation and sustained high expression of endogenous *FOXP3* in HEK293 and human Jurkat T cell lines through targeting of the core promotor, three known regulatory regions of the *FOXP3* gene (CNS1–3), and two additional regions selected through extensive bioinformatics analysis (Cage1 and Cage2).

**Results:**

Using an activator-domain fusion based dCas9 transcription activator, robust upregulation of *FOXP3* was achieved, and an optimal combination of single guide RNAs was selected, which exerted an additive effect on *FOXP3* gene upregulation. Simultaneous targeting of *FOXP3* and *EOS*, a transcription factor known to act in concert with *FOXP3* in initiating a Treg phenotype, resulted in upregulation of *FOXP3* downstream genes *CD25* and *TNFR2*. When compared to ectopic expression of *FOXP3* via plasmid electroporation, upregulation of endogenous *FOXP3* via the Cas9-based method resulted in prolonged expression of *FOXP3* in Jurkat cells.

**Conclusions:**

Transfection of both HEK293 and Jurkat cells with dCas9-activators showed that regulatory regions downstream and upstream of *FOXP3* promoter can be very potent transcription inducers in comparison to targeting the core promoter. While introduction of genes by conventional methods of gene therapy may involve a risk of insertional mutagenesis due to viral integration into the genome, transient up- or down-regulation of transcription by a CRISPR–dCas9 approach may resolve this safety concern. dCas9-based systems provide great promise in DNA footprint-free phenotype perturbations (perturbation without the risk of DNA damage) to drive development of transcription modulation-based therapies.

## Background

Forkhead box P3^+^ (*FOXP3*^+^) regulatory T cells (Tregs) represent a unique subset of lymphocytes, critical for the maintenance of immune homeostasis in response to environmental antigens and immunological self-tolerance through elimination of self-reactive T cells in the thymus and peripheral organs. Loss of or defects in Treg cell populations result in immune-tolerance breakdown and autoimmunity [1]. Tregs can affect proliferation and cytokine production of conventional T cells (Tconv) directly, via cell-to-cell contact and expression of immunosuppressive soluble factors, or indirectly, via inhibition of antigen presenting cells, depending on the location and state of the interacting cells [2]. Central Tregs predominantly reside and function in lymphoid tissues, while effector Tregs downregulate lymphoid homing molecules and are capable of migrating to peripheral lymphoid organs and tissues where they upregulate activation-induced markers, such as ICOS, GITR, CD44 and others [3].

While the molecular signature of diverse subsets of Tregs differs with respect to their location, origin and function [4, 5], a common trait of all Tregs is expression *FOXP3*, the master regulator of the Treg cell phenotype [6]. Loss-of-function mutations of the *FOXP3* gene in animal models and humans result in loss of differentiation potential into Treg cells and is responsible for highly aggressive, fatal, systemic immune-mediated inflammatory disease [5]. Many autoimmune conditions, such as type 1 diabetes, multiple sclerosis, systemic lupus erythematosus, rheumatoid arthritis and others are characterized by an imbalance between the pools of immune-suppressing Tregs and pro-inflammatory CD4+ conventional T cells [7]. Based on this concept, approaches towards specific targeting of immune cells with an aim to increase the pool of Tregs have been considered for therapy of autoimmune diseases [8–10]. The Treg pool may be enhanced either by ex vivo expansion of regulatory T cells or by induction of Tregs (iTregs) from conventional T cells. Selective expansion of autologous Tregs has proved challenging especially due to the low initial number of Treg cells in patients with autoimmune diseases and altered gene expression profiles of ex vivo propagated versus naturally occurring Tregs [11]. On the other hand, ectopic expression of *FOXP3* in naïve T cells and T cell lines via viral transduction has been shown to confer in vivo and in vitro suppressive activity towards Treg cells, demonstrating that Tconv may be reprogrammed into immunosuppressive Treg-like cells [6, 12–14]. However, viral-based transduction approaches may result in varied gene expression, epigenetic silencing, insertional mutagenesis or oncogene activation by gene integration. Transdifferentiation of conventional T cells into immunosuppressive Treg-like cells using non-insertional methods via *FOXP3* upregulation could provide an alternative approach to increase the pool of therapeutic Treg-like cells.

Due to its relatively simple design and high efficiency, the clustered regularly inter-spaced short palindromic repeats (CRISPR)-associated protein 9 (Cas9) system (CRISPR–Cas9 system) in combination with a guide RNA molecule targeting a specific DNA sequence has been successfully used for genome editing by inducing sequence-specific double-stranded DNA breaks. CRISPR–Cas9 system applications [15] have been used in gene editing, applied precision genome engineering, nucleic acid imaging in live cells, diagnostics and transcriptional regulation. In addition to editing the genome sequence, several approaches to regulate epigenetics and transcription using the CRISPR–Cas9 system have also been developed. They are based on a catalytically inactive variant of Cas9 (dCas9), which retains DNA binding activity, but does not induce a double-stranded DNA break. For example, the fragile X syndrome in neuronal cells and in mice has recently been rescued by fusing dCas9 to a demethylase TET1, which corrected transcriptional regulation of the target *FMR1* gene [16]. Epigenetic remodeling by a modified dCas9 system was also used by Liao et al. [17] to modulate transcription and to generate gain-of-function phenotypes for in vivo treatment of type 1 diabetes, kidney injury, and murine muscular dystrophy. CRISPR–dCas9 applications pertaining to the study in here employ dCas9 protein fused to various effector domains for target-specific transcriptional activation and repression [18, 19]. Various genetic screens in mammalian cells to elucidate gene function and uncover novel therapeutic approaches have been conducted using such dCas9-activator or dCas9-repressor systems [20]. A so-called CRISPR interference (CRISPRi) is based on fusions of dCas9 to a Krüppel-associated box (KRAB) domain for gene repression. For example, by combining lentiCRISPR vector with dCas9 fused to a Krüppel-associated box (KRAB) repression domain, genetic knockdowns have been obtained in neurons [21]. To achieve gene activation—an approach termed CRISPR activation (CRISPRa)–dCas9 has been fused to activator domains such as VP16, VP64 or VPR [22]. Therefore, while the CRISPR–Cas9 system was initially used mainly for gene editing, the development of the dCas9 system enabled many recent applications in the area of transcriptional regulation.

In the present study, we sought to systematically employ the dCas9 CRISPR-based method for upregulation and sustained high expression of endogenous *FOXP3* in HEK293 and human Jurkat T cell lines. The effect of dCas9-based upregulation of endogenous *FOXP3* was compared to the effects of ectopic *FOXP3* expression. To obtain robust and sustained modulation of *FOXP3* expression we analysed and targeted several known and predicted regulatory regions. *FOXP3* gene expression has been shown previously to be controlled by a core promotor region and three conserved non-coding sequences (CNS1–3). The CNS1 region is involved in TGFβ signalling and is required for generation of peripheral Tregs, CNS2 is involved in the stability of *FOXP3* expression and Treg phenotype maintenance while CNS3 has a crucial role in thymic induction of *FOXP3* expression [23]. It has been shown recently [24, 25] that an important mechanism to generate stable Tregs is demethylation of CNS2. Hypomethylation was also a proposed mechanism for the activity of CNS1 [26]. In addition, this intronic enhancer element is responsive to butyrate from commensal bacteria to provide an additional molecular cue in potentiation of peripheral Treg development [27]. The third intronic regulatory site CNS3 has also been shown to be under epigenetic control [28], not via methylation, but by modifying accessibility to chromatin brought about by a long noncoding RNA *Flicr* [29]. Another study demonstrated that CNS3 is bound by an atypical inhibitor of NF kappa B (I kappa B), I kappa B-NS, resulting in changes of *FOXP3* expression and regulation of Treg cell development [30]. Apart from targeting these previously studied intronic regulatory sites, we also performed a bioinformatics analysis to identify additional regulatory sites in the *FOXP3* gene. On the basis of a previous genome-wide study using the method of cap analysis of gene expression (CAGE) two more active enhancer elements specific for Tregs were identified and found to be very potent transcription inducers when targeted by designed sgRNA in combination with the dCas9-activator system. In all aforementioned regulatory sites, we designed several variants of single-guide RNAs (sgRNAs) and assessed expression of genes, known to signal downstream of *FOXP3*. Upregulation of *FOXP3* via dCas9-activator was compared to ectopic expression of *FOXP3*. We systematically tested the effects of targeting either individual or multiple regulatory regions to define a combination of sgRNAs with a maximal effect on *FOXP3* upregulation.

## Results

### Identification of *FOXP3* regulatory regions and sgRNA design

**Fig. 1:**
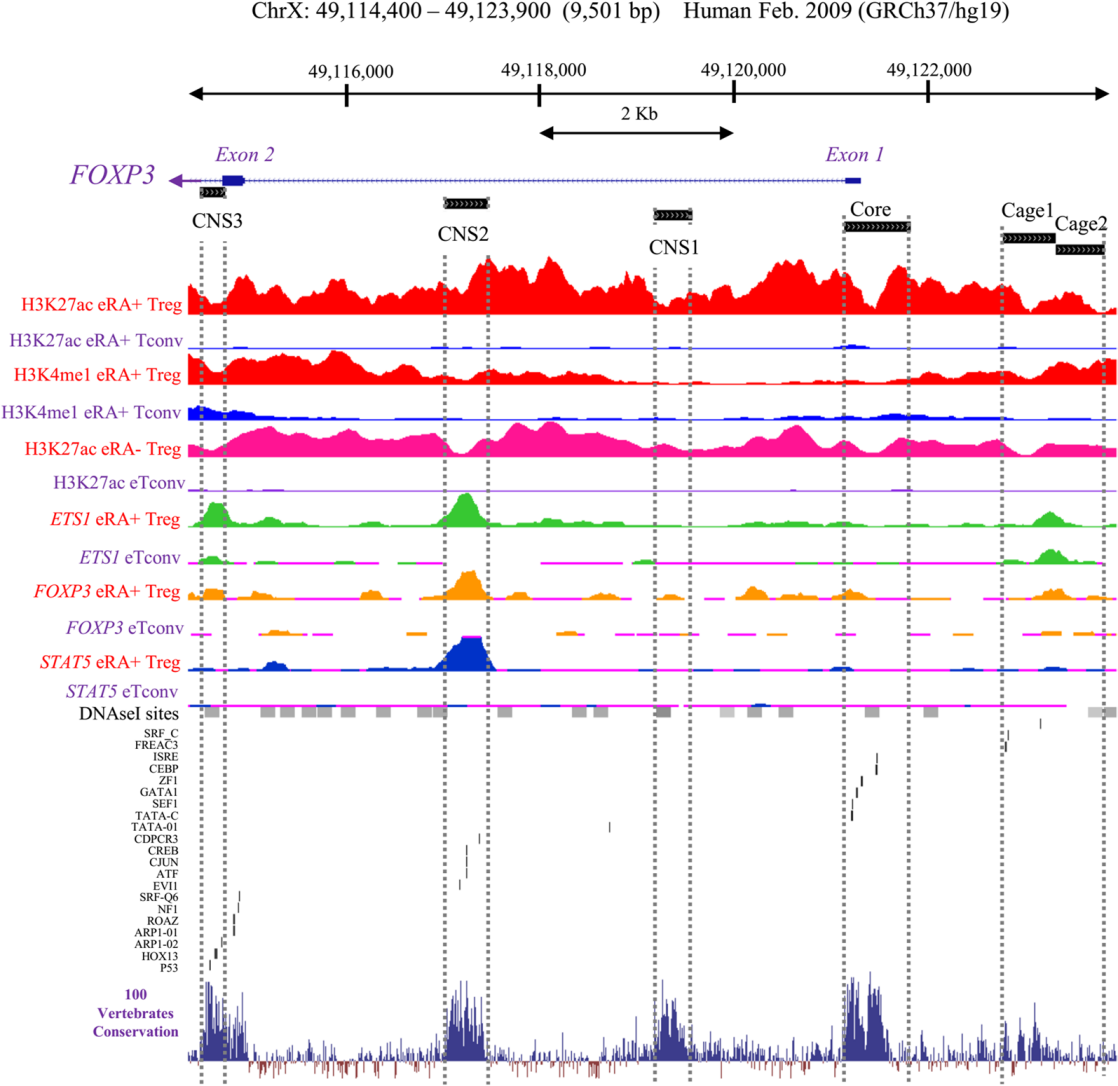
Bioinformatics analysis for identification of regulatory sites in the *FOXP3* gene for targeting by a CRISPR. The genomic segment encompassing the human *FOXP3* locus on chromosome X (49,114,400–49,123,900 GRCh37/hg19 assembly) is shown. Regions named CNS1–3, Core promoter, Cage1 and Cage2 were identified (vertical broken gray lines) and targeted by sgRNA–dCas9VPR or sgRNA-dCas9-KRAB fusions. UCSC browser (https://genome.ucsc.edu) default graphics have been modified and customized tracks pertinent to our study were included. Targeted regions were selected based on the literature review of *FOXP3* regulatory sites and if differential regulatory activity of *FOXP3* upstream and downstream sequences in Treg vs. Tconv cells existed [11]. Cell types are: CD4+CD25highCD45RA+naïve (eRA+ Treg) and in vitro expanded CD4+ CD25-conventional T cells (eTconv). Additionally, *FOXP3*, ETS1, and STAT5 cell type-specific enhancer architecture and gene regulation by chromatin immunoprecipitation sequencing (ChIP-seq), differential epigenetic modifications such as histone H3K27 acetylation (H3K27ac) and H3K4 methylation (H3K4me1) signals, DNaseI hypersensitive sites, conserved transcription binding sites and 100-vertebrates basewise conservation segments are shown to be clustered in these targeted regions (more details on http://www.ag-rehli.de/NGSdata.htm, by following the link “HeliscopeCAGE, H3K27ac, and H3K4me1 as well as *FOXP3*, STAT5, ETS1 and RUNX1 ChIPs for T cell subpopulations

Bioinformatic analysis for identification of the target regulatory sites in the *FOXP3* gene was performed using published experimental [31] and prediction data from bioinformatics databases [32]. In particular, binding sites for transcription factors and RNA polymerases, open chromatin, histone modifications, CpG islands, TATA-boxes, genetic variability, conserved sequences between species and within the human genome were analysed to identify the top candidate regulatory sites for CRISPR-mediated *FOXP3* gene targeting. Ensembl genome database [33] and tools were used to identify evolutionary constrained elements based on the following criteria; (a) presence of at least one evolutionary conserved element (b) a minimum of two other regulatory features such as open chromatin, transcription factor binding site, histone modification, RNA polymerase binding site, DNA methylation, CpG island or micro RNA (miRNA) binding sites. In addition to known regulatory regions of the *FOXP3* gene (CNS1–3) [23], two additional regions, termed Cage1 and Cage2, were selected for targeting in the 5′ upstream of the *FOXP3* core promoter (Fig. 1). Regions Cage1 and Cage2 were chosen based on a study of Schmidl et al. [11] and data on http://www.ag-rehli.de/NGSdata.htm that demonstrated regulatory activity on these upstream sequences in Treg cells. The Eukaryotic promotor database was employed to determine the core promoter region of *FOXP3* at − 511/+ 176 relative to the transcription start site. Specifically, the core promoter region encompassed 49,264,651–49,265,336 bp region on human chromosome X (ChrX), three regions downstream of core promoter were CNS1 (ChrX: 49,262,712–49,263,103 bp), CNS2 (ChrX: 49,260,567–49,261,013 bp) and CNS3 (ChrX: 49,258,069–49,258,320 bp) and two regions upstream of core promoter entailed Cage1 (ChrX: 49,266,272–49,266,819 bp) and Cage2 (ChrX: 49,266,819–49,267,319 bp; all above mentioned coordinates pertain to the human genome assembly hGRC37/hg19). Together, six regions were determined as targets for *FOXP3* gene regulation including the core promotor, CNS1, CNS2, CNS3, Cage1 and Cage2 (Fig. 1). Several sgRNA variants (Table 1) were designed to target each of the regions using Benchling Biology Software [34].

**Table 1:** sgRNA target sequences used for *FOXP3* gene upregulation

Regulatory region	sgRNA target sequence	sgRNA
Core	tgtgtgcgctgataatcacg	Fox1
	tgcttgaactacccggcgag	Fox2
	cattgcttgaactacccggc	Fox3
	tatagatggaattgatatgg	Fox4
CNS1	atagggcttggggtgacgct	Fox5
	aaaatcacacatagggcttg	Fox6
	gtacccacactcttaacctc	Fox7
	agacagtctggctccagtac	Fox8
CNS2	tcatggcggccggatgcgcc	Fox9
	cagattatgttttcatatcg	Fox10
	gatgcgccgggcttcatcga	Fox11
	caccccacaggtttcgttcc	Fox12
CNS3	aggtcggcacctgtaggtcc	Fox13
	agacagggattgggaggtcg	Fox14
	cagtaaaggtcggcacctgt	Fox15
	taacagatgtcacggcatgt	Fox16
Cage1	caagactggcttcagacctg	Fox17
	aaccttctaagccctcgtaa	Fox18
	caccattagttcaaaacaaa	Fox19
	ctcctgcgtaattataaacc	Fox20
Cage 2	tataggaggcaaacgaagtg	Fox21
	gcgtaattataaaccaggcc	Fox22
	tgcagacttgggtcggaatg	Fox23
	tacacctcctgcgtaattat	Fox24

### Targeting of *FOXP3* regulatory regions using a CRISPR–dCas9 based system in HEK293 cells

**Fig. 2:**
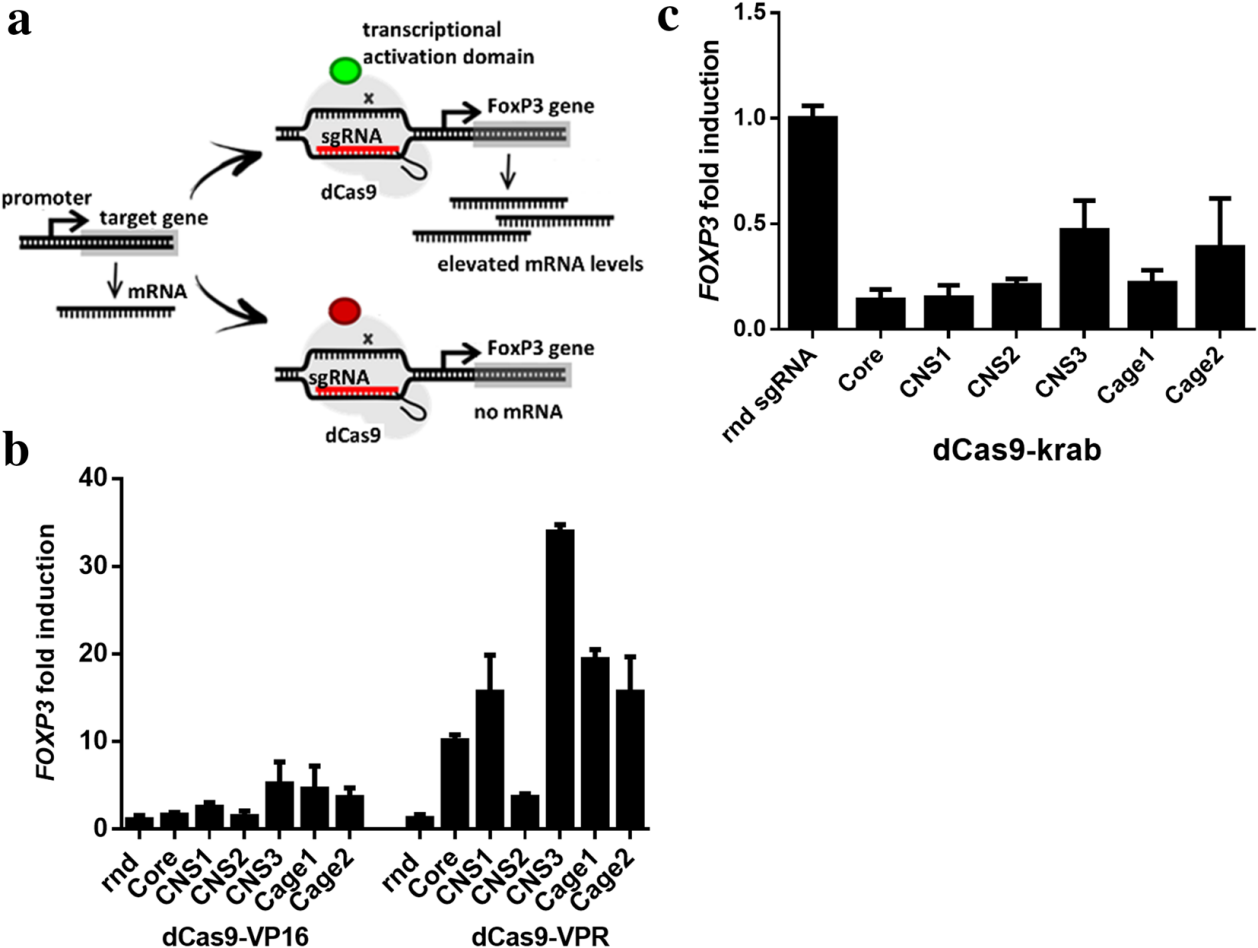
Targeted up and down-regulation of the *FOXP3* transcript levels in HEK293 cells. **a** Schematic presentation of the transcriptional regulation with CRISPR–dCas9. **b**, **c** HEK293 cells were transfected with plasmids encoding sgRNAs targeting six distinct regulatory regions of the *FOXP3* gene (see Fig. 1) and cotransfected with plasmids encoding dCas9-based activators (VP16 or VPR) or a repressor (KRAB). Fold expression of *FOXP3* was analyzed in respect to a control sample transfected with a random sgRNA (rnd) and normalized to a house-keeping gene (GAPDH). A mixture of four sgRNAs was used for each region (sgRNA sequences are listed in Table 1). RNA was isolated from samples 48 h post transfection. Experiments were repeated 3 times. b Results using dCas9-VP16 or VPR activators show that regulatory target sites downstream (CNS3, CNS1) and upstream (Cage, Cage2) of core promoter can result in higher activation of *FOXP3* transcription than sgRNA targeting core promoter. **c** Results using dCas9-KRAB demonstrate that *FOXP3* basal transcription can be further downregulated using fusion of dCas9 with a repressor domain KRAB

Different fusion variants of the catalytically inactive Cas9 (dCas9) fused to transcriptional activation or repression domains were tested in combination with sgRNA molecules targeting indicated regions with the goal of inducing robust up or down-regulation of *FOXP3* expression (Fig. 2a). sgRNAs, targeting the core, Cage or CNS regions, upstream or downstream of the *FOXP3* core promoter coding sequence, were transfected into HEK293 cells in combination with plasmids encoding dCas9-based activators (VP16 or VPR) or a repressor (KRAB). Each distinct region was targeted using a combination of four sgRNA variants in combination with the dCas9-VPR activator.

Interestingly, upregulation through targeting the core promotor region did not result in the highest levels of *FOXP3* upregulation with both of the dCas9-based activators. Using dCas9-VPR activator, ranking of the highest to lowest transcription induction effects of *FOXP3* was as follows: the CNS3 (~ 35-fold), Cage1 (~ 20-fold), Cage2 and CNS1 (~ 15-fold), core promoter (~ 10-fold) and CNS2 (~ 4-fold) (Fig. 2b). Therefore, targeting intronic and upstream enhancer regions resulted in approx. 2 to 3.5-fold higher induction of *FOXP3* expression compared to targeting of the core promotor region. Overall, the dCas9-VPR transcription activator resulted in about one order of magnitude higher levels of *FOXP3* upregulation in comparison to the dCas9-VP16 activator (Fig. 2b). The latter was therefore selected for further experiments addressing *FOXP3* upregulation. Additionally, a dCas9-based repressor, dCas9-KRAB, was tested for downregulation of *FOXP3* gene expression. Cells, transfected with plasmids encoding selected sgRNA constructs in combination with dCas9-KRAB exhibited a reduced level of *FOXP3* expression in comparison with cells, transfected with a control sgRNA (Fig. 2c). Targeting CNS3 and Cage2 repressed transcription by about 50% while core, CNS1, CNS2 and Cage1 repressed transcription by 70–80%.

### Targeting of *FOXP3* regulatory regions using a CRISPR–dCas9 based system in Jurkat T cells

**Fig. 3:**
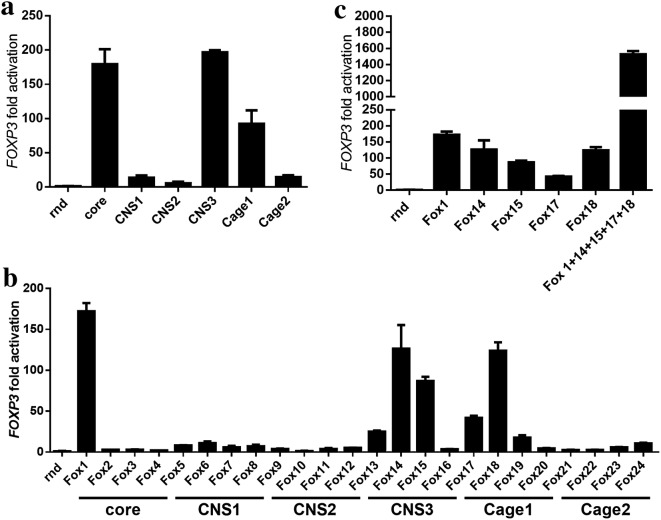
Upregulation of *FOXP3* mRNA in Jurkat cells via targeting of selected promoter and enhancer regions. Jurkat cells were electroporated with plasmids encoding indicated single sgRNAs or a combination of sgRNAs in combination with the dCas9-VPR transcriptional activator and fold activation of *FOXP3* gene expression was measured in reference to a control sample, electroporated with plasmids encoding random sgRNA (rnd) and dCas9-VPR. Each experiment was repeated 3 times. Cells were lysed and RNA isolated 48 h post electroporation. **a** Jurkat cells were electroporated with a combination of four sgRNAs targeting each region [Core (Fox 1–4), CNS1 (Fox 5–8), CNS2 (Fox 9–12), CNS3 (Fox 13–16), Cage1 (Fox 17–20) and Cage2 (Fox 21–24)] and *FOXP3* gene expression was measured. **b** Jurkat cells were electroporated with each sgRNA (Fox 1–24) separately and the effect of each single sgRNA on *FOXP3* gene expression was measured. **c** Jurkat cells were electroporated with sgRNAs 1, 14, 15, 17 and 18 separately and in combination and *FOXP3* gene expression was measured

Upregulation of *FOXP3* through targeting of six distinct regulatory regions was further characterized in Jurkat cells, a more relevant immune cell line derived from leukemic T cell lymphoblasts. Each distinct region was targeted using a combination of four sgRNA variants. While upregulation through targeting CNS3 resulted in a robust upregulation of *FOXP3* in both cell lines, targeting other regions differed somewhat between HEK293 and Jurkat cells (Figs. 2b and 3a). The contribution of each single sgRNA was tested and the results used to select an optimal combination of several sgRNAs for an optimal effect on *FOXP3* upregulation (Fig. 3b). The most efficient single variants [sgRNAs targeting the core (sg1), CNS3 (sg14, 15) and Cage1 (sg17, 18) regions] were combined which showed an additive effect on *FOXP3* upregulation (Fig. 3c). Induction of *FOXP3* in HEK293 and Jurkat cells showed comparable results with several, but not all selected sgRNAs. These findings suggest that selected sgRNAs, targeting *FOXP3* regulatory elements CNS3, Cage1 and the core promoter region significantly and robustly activate expression of the *FOXP3* gene and can exert an additive effect on transcription when combined.

### Robust and prolonged endogenous *FOXP3* upregulation in Jurkat T cells using the CRISPR–dCAS9-VPR system

**Fig. 4:**
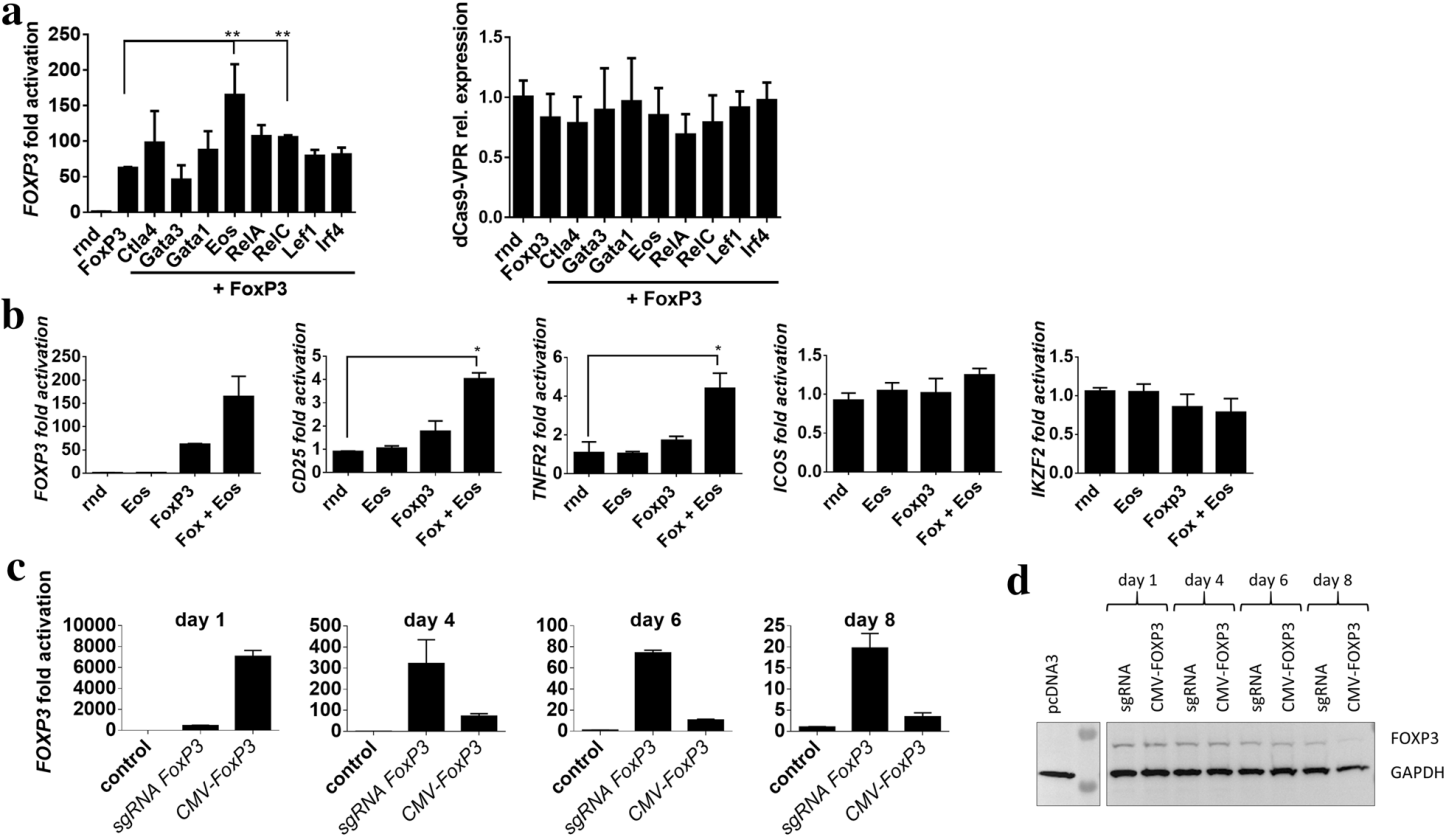
Robust and prolonged endogenous *FOXP3* upregulation in Jurkat cells. **a**, **b** Jurkat cells were electroporated with plasmids encoding control sgRNA (rnd), sgRNA for *FOXP3* upregulation (Fox 1, 14, 15, 17 and 18; sequences in Table 1), sgRNA for *EOS* upregulation, sgRNA for *FOXP3* upregulation in combination with an additional candidate gene (*CTLA4*, *GATA3*, *GATA1*, *EOS*, *RELA*, *RELC*, *LEF1*, *IRF4*; sequences listed in Table 2) and fold activation of exogenic *FOXP3*, *Cas9*, *CD25*, *TNFR2*, *ICOS* and *IKZF2* were measured. Experiments were repeated 3 times. Cells were lysed and RNA isolated 48 h post electroporation **c**, **d** Jurkat cells were electroporated with either a plasmid encoding *FOXP3* under the control of a CMV reporter (*CMV*-*FOXP3*) or plasmids for upregulation of endogenous *FOXP3* via CRISPR–dCas9 (sgRNA *FOXP3*) or a pcDNA3 control vector. Fold activation and protein expression were measured on days 1, 4, 6 and 8 post electroporation

Previous studies have demonstrated that the expression of ectopic *FOXP3* in addition to other transcription factors (e.g. *GATA*-*1*, *EOS*, *IRF4* and others) can shift the transcriptional signature towards a Treg phenotype due to enhancement of occupancy of *FOXP3* at its genomic target sites [35, 36]. We tested upregulation of several additional genes, typically expressed in T regulatory cells in a pairwise combination with *FOXP3* to test for additional increase in expression of *FOXP3* and downstream gene targets of *FOXP3*, such as *CD25*, *TNFR2, ICOS* and *IKZF2* (Fig. 4a, b). Interestingly, a pairwise combination of *FOXP3* and *EOS* targeting via sgRNA and dCas9-VPR further increased the expression levels of *FOXP3*, while no change in *FOXP3* expression was noted when combined with upregulation of other candidate genes (Fig. 4a, left). Expression of dCas9-VPR, relative to a control sample transfected with dCas9-VPR in combination with a random sgRNA was measured. Stable expression levels of dCas9-VPR indicated that the increase was in fact due to additional upregulation of *EOS* or *RELC* and not variability in dCas9-VPR expression between samples (Fig. 4a, right). sgRNA dCas9-VPR targeting of *FOXP3* and *EOS* was further tested for the effect on genes, upregulated downstream of *FOXP3* in Tregs. While upregulation of *FOXP3* or *EOS* separately showed no upregulation of downstream genes, simultaneous targeting of both resulted in increased expression levels of *CD25* and *TNFR2*, while *ICOS* and *IKZF2* expression levels remained constant in all cases (Fig. 4b).

To compare the CRISPR–dCas9-based method of *FOXP3* upregulation to ectopic *FOXP3* expression, we electroporated Jurkat cells with a plasmid encoding *FOXP3* under the control of a constitutive CMV promotor or plasmids encoding sgRNA for *FOXP3* upregulation in combination with the dCas9-VPR designed transcription factor. *FOXP3* gene expression was measured at several time-points (1, 4, 6, and 8 days) post electroporation. Interestingly, *FOXP3* expression levels remained fairly stable for the first 4 days post electroporation (approx. 500 and 400-fold above control samples on days 1 and 4, respectively) when *FOXP3* was upregulated via dCas9-VPR and decreased by days 6 and 8 post electroporation, although still expressed around 20-fold above the control sample on day 8 (Fig. 4c). On the other hand, in the case of ectopic *FOXP3* expression, levels ranged up to several thousand-fold above control on day 1, but rapidly dropped on consecutive days well below levels of endogenous *FOXP3*, upregulated via CRISPR–dCas9 (Fig. 4c). Western blot analysis showed that *FOXP3* protein expression was in agreement with mRNA expression assays, although differences in protein expression were somewhat more modest than differences noted in mRNA levels on consecutive days (Fig. 4d). These results point to a preference for upregulation of endogenous *FOXP3* via CRISPR–dCas9, especially when the goal is prolonged upregulation.

## Discussion

It has become evident over the past decade that the immune system is under strong control mediated by specialized cell subsets that suppress immune reactivity, with the CD4+CD25+*FOXP3*+ Treg subset representing the most prominent of the immunosuppressive cells. *FOXP3* expression is crucial throughout the Treg cell life-time to sustain a Treg phenotype and prevent autoimmunity and defects in *FOXP3* expression, which have been associated with severe autoimmune conditions [7]. Autologous transfer of T regulatory cells has been used in treating diverse autoimmune conditions. Several studies have shown that induction of *FOXP3* expression enables the differentiation of Tregs from conventional CD4+ T cells [6, 12–14]. This approach may be superior to Treg cell isolation and expansion, since the percentage of CD4+ Tconv cells in the human blood is several orders of magnitude larger (10–20% of leukocytes) compared to the pool of Treg cells (only 0.1–0.7%). In autoimmune patients, the frequency of Tregs is depleted even more and hence isolation of sufficient therapeutic doses of natural Tregs even by expansion is problematic. A possible solution represent induced Treg (iTreg) cells, but several issues of ex vivo-expanded iTregs remain to be resolved prior to the clinical application, since iTregs can convert to other pathogenic T cell subsets when in an in vivo environment [37].

In previous studies on Treg phenotype induction, *FOXP3* was expressed ectopically or transduced via viral vectors to cells [6, 12–14]. In the present study, we sought to upregulate *FOXP3* via a CRISPR–dCas9-based method as an alternative approach to viral-based methods and to compare upregulation of endogenous to ectopically expressed *FOXP3*. As a result of a comprehensive bioinformatics analysis, described in the results, six regions likely to contain important *FOXP3* regulatory features, were identified within the promotor and enhancer regions of the *FOXP3* locus (the core promotor region, CNS1, CNS2, CNS3 and Cage 1 and 2), and targeted using a system of dCas9 fused to activator(s) or a repressor. Using a combination of sgRNAs designed to target the selected regulatory regions, we were able to achieve robust up- or down-regulation of *FOXP3* expression in HEK293 cells using a transcriptional activator (dCas9-VPR) or repressors (dCas9-KRAB), respectively.

While introduction of genes by conventional methods of gene therapy for the overexpression of transcription factors may involve a risk of insertional mutagenesis due to viral integration into the genome, transient up- or down-regulation of transcription by a CRISPR–dCas9 approach may resolve this safety concern. dCas9-based systems provide great promise in DNA footprint-free phenotype perturbations (perturbation without the risk of DNA damage) to drive development of transcription modulation-based therapies.

Five sgRNAs targeting each of the regulatory regions that most efficiently induced transcription in preliminary tests were transfected into HEK293 cells or Jurkat cells in combination with the dCas9-VPR transcription activator. Each of the single sgRNAs targeting a specific regulatory region was separately tested for its effect on *FOXP3* induction. Based on the results of individual sgRNAs, an optimal combination of the designed sgRNAs was selected to exert an effect on *FOXP3* upregulation. While most of the targeted regions exhibited a significant effect on *FOXP3* induction, targeting of CNS3, CNS1, Cage1 and Cage2 regions showed a significantly higher effect on *FOXP3* upregulation than targeting the core promoter in HEK293 cells. In Jurkat cells, targeting CNS3 and Cage1 also proved to be most effective in *FOXP3* induction, but targeting the core promoter also elicited a robust response. In different cell types, regulatory regions can be differentially epigenetically modified and thus differentially accessible for transcription factor binding. Differential chromatin state in the utilized cell lines could be one of the underlying causes explaining differential effect of different sgRNAs on HEK293 and Jurkat cells.

Transfection of both HEK293 and Jurkat cells with dCas9-activators showed that regulatory regions downstream and upstream of the *FOXP3* promoter can be very potent transcription inducers in comparison to targeting the core promoter. Upon detailed literature and bioinformatics analyses, we identified several candidate regulatory sites in the *FOXP3* gene outside the core promoter. The important role of intronic conserved non-coding sequence (CNS) elements CNS1–3 in defining the fate and stability of the Treg cells was already demonstrated [23]. In our study targeting each of the three intronic enhancer elements (CNS1–3) resulted in *FOXP3* induction comparable or higher to targeting the core promoter, whereas CNS3 showed the highest and the most robust effect of all the treatments in both tested cell lines. Results of our study therefore confirmed the importance and potency of intronic (CNS1–3) elements in transcriptional control of *FOXP3*.

Furthermore, our study is to our knowledge the first to demonstrate the efficiency of sgRNA-targeting of *FOXP3* distal enhancer regulatory sites, Cage1 and Cage2, for dCas9-VPR transcriptional control of this gene. These two sites were previously identified in a genome-wide cataloguing of the marks associated with active chromatin in Tregs [11]. Targeting Cage1 and Cage2 enhancer sites with dCas9-fused activators proved efficient in activating *FOXP3* expression. Although Cage1 and Cage2 are located about 1500 bp and 2000 bp upstream of the core promoter, respectively, our study identifies these two noncoding sites as very potent regulatory regions for *FOXP3* transcription modulation.

Most studies [24] using dCas9-activator system for modulation of target gene expression tend to target the core-proximal promoters with sgRNA. This is most likely because the web-based tools to design sgRNA essentially always target core promoters usually around − 400 to + 50 or less. This is understandable as each gene can have various active regulatory target sites located up or downstream of the gene, most of which are yet not known, or are not properly annotated. If anything, our study suggests, that for more efficient gene regulation, one may need to first identify, on a gene-by-gene basis, potential regulatory sites outside the core promoter and experimentally target them with sgRNA to compare efficiency with targeting core promoter only. These distal regulatory regions can be especially important when experimental objective is cell- or tissue-specific transcription modulation. Also, as in our case, additive or synergistic effects can be expected when targeting multiple sites in the promoter and enhancers instead of or in addition to the core promoter.

Stable and high expression of *FOXP3* is associated with positive feedback loops of *FOXP3* in coordination with several other transcription factors. We noted that combined upregulation of *FOXP3* and *EOS* resulted in higher fold of *FOXP3* expression than upregulation of *FOXP3* alone. While *EOS* has been shown to be redundant for Treg development, it has been implicated in controlling many functions of Tregs, promoting Treg survival and increasing occupancy of *FOXP3* at its genomic targets, the latter role presenting a possible explanation for the observed increase in *FOXP3* upregulation [36, 38, 39].

Furthermore, we demonstrated that *FOXP3*-*EOS* upregulation resulted in an increase in *CD25* and *TNFR2* expression, known to function downstream of *FOXP3* in the Treg signalling network. This is in line with previous evidence demonstrating that *CD25* is directly regulated by *FOXP3* via regulatory region on *CD25* promoter [40] although upregulation of *FOXP3* alone did not cause this effect in our case. Additionally, the observed *TNFR2* upregulation is supported by experiments showing that a combined higher *CD25* and *TNFR2* expression is of paramount importance for phenotypic and functional stability of Tregs [41, 42]. Another group found that a subset of Treg cells with the highest *CD25* and *TNFR2* expression also exhibited maximal proliferative and effector cytokine-producing capability [43]. Reproduction of a full Treg transcriptome and phenotype was not expected in our case, as the Jurkat T cell line is not likely to fully reproduce all characteristics of primary T cells. Nevertheless, treatment of Jurkat cells with dCas9-VPR activator targeting *EOS* and *FOXP3* shifted the transcriptional profile into a *FOXP3*+/CD25+/TNFR2+ state characteristic of stable and functional Treg primary cells.

While also *FOXP3*-independent mechanisms are most likely required to obtain full suppressive function of Tregs, stable expression of *FOXP3* is a prerequisite in Treg induction and function. A CRISPR-based method of endogenous *FOXP3* upregulation proved to be superior to ectopic expression of *FOXP3* via plasmid transfection in terms of prolonged expression. Further studies with recombinant dCas9-VPR fusion protein and sgRNA delivery will be required to test our system in primary human T cells and in vivo. Although historically primary T cells proved refractory to efficient non-viral transfection protocols, there are recent developments of efficient electroporation methods also for primary T cells [44]. This study provided promising results in electroporation efficiency of an ssDNA and Cas9–sgRNA ribonucleoprotein (RNP) complex in comparison with double stranded DNA without detectable toxicity and off-target effects. In conclusion, the identified regulatory target sites in the *FOXP3* gene in our study represent a useful addition to the Treg phenotype induction toolkit and may be used in combination with other known approaches, such as epigenetic modifications of *FOXP3* regulatory loci [45], TGFβ stimulation and others [46].

## Methods

### Plasmids and cloning

All guide RNAs were cloned via PCR into the pgRNA-humanized vector harboring the U6 promotor for expression in mammalian cells (Addgene plasmid 44248). gRNAs were designed in silico using Benchling Biology Software [34] and are listed in Tables 1 and 2. dCas9 was obtained from pHR-SFFVdCas9-BFP-KRAB (Addgene plasmid 46911). The VPR, VP16 and KRAB activator/repressor sequences were synthesized by Genewiz and cloned with Gibson assembly method into the pCMV-dCas9-VPR vector. The human *FOXP3* gene sequence was cloned into the pcDNA3 vector. For control, pcDNA3 (Invitrogen) was used.

**Table 2:** sgRNA target sequences

Target gene	sgRNA target sequence
*CTLA4*	cgacgtaacagctaaaccca
*GATA3*	ccgcagagggcggccgccgg
*GATA1*	gcgaggtccaagaatcccca
*EOS (IKZF4)*	gcataccagacacataggag
*RELA*	gccccgccgccgcccggcgc
*RELC*	tcgcgcgcgcggcggccgcg
*LEF1*	tccttggctgcccgctggag
*IRF4*	cgctctccgggcgcggcgcg

### Reagents

The following primary antibodies were used for western blotting: Mouse monoclonal to *GAPDH* diluted 1:2000, purchased from Proteintech (#60004-1-Ig), Mouse monoclonal [236A/E7] to *FOXP3* from Abcam (1:1000) and Goat Anti-Mouse IgG (H+L)-HRP diluted 1:3000 (Jackson ImmunoResearch #115-035-003).

### Cell cultures

The human embryonic kidney (HEK) 293 and Jurkat cell lines were purchased from American Type Culture Collection (ATCC). Cells were cultured in DMEM (Invitrogen Life Technologies) supplemented with 10% (v/v) heat-inactivated FBS (Invitrogen Life Technologies) (HEK293) or RPMI (Invitrogen Life Technologies), supplemented with 10% (v/v) heat-inactivated FBS (Invitrogen Life Technologies) (Jurkat) at 37 °C in 5% CO_2_.

### Cell transfection and electroporation

HEK293 cells were seeded in 6- or 12-well plates (Corning, NY, USA) at 2.2 × 10^5^ cells per ml in 2 ml of media (4.4 × 10^5^ cells per well). The next day, cells were transiently transfected with plasmids using the jetPEI transfection reagent (Polyplus Transfection, NY). The total amount of DNA for each transfection was kept constant by adding appropriate amounts of pcDNA3 plasmid (Invitrogen). Jurkat cells were electroporated with the Neon Transfection system (Thermo Fisher Scientific) at 1350 V, 10 ms, 3 puls in R buffer as per manufacturer’s protocol. A total of 10–15 μg of DNA was used to electroporate 2 × 10^6^ cells for each sample. Cells were electroporated at 2 × 10^7^ cells per ml in 100 μl electroporation buffer and seeded in 2 ml of media. The total amount of DNA for each transfection was kept constant by adding appropriate amounts of control plasmid. Each experiment was repeated at least three times, and each measurement was performed in at least 2 parallels.

### RNA isolation and cDNA synthesis

Total RNA was extracted (at least) 48 h (or else indicated) post electroporation/transfection from Jurkat or HEK293 cells using the High Pure RNA Isolation kit (Roche). RNA quantity and purity was measured on Nanodrop spectrophotometer (Thermo Fisher Scientific). The RNA samples were diluted to a final concentration of approximately 100 ng/µl total RNA. The diluted RNA was treated with DNase I (Thermo Fisher Scientific) and reverse transcribed into cDNA, using High Capacity Reverse Transcription cDNA kit (Thermo Fisher Scientific) according to the manufacturer’s instructions.

### Reverse transcription quantitative PCR (RT-qPCR)

Relative expression of target genes (*CD25*, *dCas9*, *FOXP3*, *GITR*, *ICOS*, *IKZF2*, *IRF4*, *PI16*, *PTPRC*, *TNFR2*) was determined using reverse transcription quantitative polymerase chain reaction (RT-qPCR). The expression levels of target genes were normalised with glyceraldehyde-3-phosphate dehydrogenase (*GAPDH*) (reference gene). All primers used for qPCR are listed in Table 3. The RT-qPCR was performed on an ViiA7 real-time PCR system (Thermo Fisher Scientific). All qPCR reactions were performed in triplicate and contained 2× PowerUp SYBR Green PCR master mix (Thermo Fisher Scientific), water, 0.5 µM of each primer, and cDNA in a total volume of 10 µl. The thermal cycling conditions were the following: 2 min at 50 °C (UDG activation), 10 min at 95 °C (polymerase activation and initial denaturation), and 40 cycles at 95 °C for 15 s (denaturation) and at 60 °C for 1 min (annealing and extension). Melting curve analysis (15 s at 95 °C, 1 min at 58 °C, and 15 s at 95 °C), no-template (NTC) and no-reverse transcriptase (no-RT) controls were performed in every run to monitor potential nucleic acids contamination and primer dimer formation.

**Table 3:** RT-qPCR primer sequences

Gene	Forward (5′−> 3′)	Reverse (5′−> 3′)	RefSeq accession number
*CD25*	cgcagaataaaaagcgggtca	acttgtttcgttgtgttccga	NM_000417.2
*dCas9*	ggatcgaagagggcatcaaa	gttcctggtccacgtacatatc	KR011748.1
*FOXP3*	gcaccttcccaaatcccagt	ggccacttgcagacacca	NM_014009.3
*GAPDH*	tgcaccaccaactgcttagc	ggcatggactgtggtcatgag	NM_002046.7
*GITR*	ccagtgtatcgactgtgcctcg	cacagcgttgtgggtcttgttc	NM_004195.3
*ICOS*	cccataggatgtgcagcctttg	ggctgtgttcactgctctcatg	NM_012092
*IKZF2*	acactctggagagaagccgttc	ccagtgaactgcgctgcttgta	NM_016260.3
*IRF4*	gaacgaggagaagagcatcttcc	cgatgccttctcggaactttcc	NM_002460.4
*PI16*	ctggtgtgcaactatgagcctc	ggcaaatcctgagcatcttccg	NM_153370.3
*PTPRC*	cttcagtggtcccattgtggtg	ccactttgttctcggcttccag	NM_002838.5
*TNFR2*	cgttctccaacacgacttcatcc	acgtgcagactgcatccatgct	NM_001066.3

The comparative C_T_ method (∆∆C_T_ method) [47] was used to determine differential gene expression between treated (induction of target genes with different sgRNA–dCas9 complexes) and calibrator (untreated) samples. Primer efficiency was determined for all the primer pairs over a six-log cDNA dilution points. All the determined efficiencies (E = 10^−1^/^slope^) were found to be in the range of 100 ± 10% (R^2^ ≥ 0.99). Additionally, assay validation experiments were performed for individual target-reference primer pairs to determine if the primer efficiencies of the target and the reference are approximately equal. Normalised C_T_ values of the target genes in different template dilution points were plotted vs. log input RNA amount to create a semi-log regression line. The slopes of the validation lines (∆C_T_ vs. log input) were < 0.1 for all the primer pairs.

### Immunoblotting

Samples were lysed in passive lysis buffer (Promega). Proteins were separated by SDS-PAGE and transferred to a Hybond ECL nitrocellulose membrane (GE Healthcare). Blots were incubated with appropriate antibodies by the use of iBind Western Systems (ThermoFisher Scientific) according to the manufacturers’ protocol. The immunoblots were visualized on G-box (Syngene) after they were developed using Pico or Femto Sensitivity substrate (ThermoFisher Scientific).

### Statistical analyses

Data are presented as mean ± SD or ± SEM. Representative graphs and images are shown. A one-way anova test was used for statistical comparison of the data using GraphPad Prism version 6.00 for Windows, GraphPad Software, San Diego California USA, http://www.graphpad.com.

## Data Availability

The data supporting the conclusions of this article are included within the article.
